# Discovery, Yield Improvement, and Application in Marine Coatings of Potent Antifouling Compounds Albofungins Targeting Multiple Fouling Organisms

**DOI:** 10.3389/fmicb.2022.906345

**Published:** 2022-07-07

**Authors:** Weiyi She, Wei Ye, Aifang Cheng, Wenkang Ye, Chunfeng Ma, Ruojun Wang, Jinping Cheng, Xuan Liu, Yujing Yuan, Sin Yu Chik, Jessie James Limlingan Malit, Yanhong Lu, Feng Chen, Pei-Yuan Qian

**Affiliations:** ^1^Southern Marine Science and Engineering Guangdong Laboratory (Guangzhou), Guangzhou, China; ^2^Department of Ocean Science and Hong Kong Brach of Southern Marine Science and Engineering Guangdong Laboratory (Guangzhou), Hong Kong University of Science and Technology, Hong Kong, China; ^3^SZU-HKUST Joint PhD Program in Marine Environmental Science, Shenzhen University, Shenzhen, China; ^4^Faculty of Materials Science and Engineering, South China University of Technology, Guangzhou, China; ^5^Institute for Advanced Study, Shenzhen University, Shenzhen, China

**Keywords:** antifouling activity, marine coatings, albofungin yield improvement, *Streptomyces chrestomyceticus*, cluster-situated regulator

## Abstract

Marine biofouling caused huge economic losses of maritime industries. We aim to develop high-efficient, less-toxic, and cost-effective antifoulants to solve the problems of biofouling. In this study, we described the antifouling compounds albofungin and its derivatives (albofungin A, chrestoxanthone A, and chloroalbofungin) isolated from the metabolites of bacterium *Streptomyces chrestomyceticus* BCC 24770, the construction of high-yield strains for albofungin production, and application of albofungin-based antifouling coatings. Results showed that these albofungins have potent antibiofilm activities against Gram-positive and Gram-negative bacteria and anti-macrofouling activities against larval settlement of major fouling organisms with low cytotoxicity. With the best antifouling activity and highest yield in bacterial culture, albofungin was subsequently incorporated with hydrolyzable and degradable copolymer to form antifouling coatings, which altered biofilm structures and prevented the settlement of macrofouling organisms in marine environments. Our results suggested that albofungins were promising antifouling compounds with potential application in marine environments.

## Introduction

Marine biofouling is the natural colonization of undesirable micro and macro-organisms on submerged artificial surfaces. These organisms are roughly bifurcated into microfoulers such as bacteria and diatoms and macrofoulers such as barnacles, mussels, tubeworms, and bryozoans (Callow and Callow, 2002). The bacterial biofilm is composed of diverse bacterial colonies of cells that form a community. It is a multidimensional (3-D) aggregation of bacteria attached to a surface and enclosed in an extracellular polymeric matrix, which is composed of polysaccharides, proteins, environmental DNA, and phospholipids (Srinivasan et al., 2021). The colonization through biofilm formation by microfoulers attracts macrofoulers to attach, such as the tubeworm *Hydroides elegans*, which prefer to settle on biofilm surfaces (Ralston and Swain, 2009). Compared to planktonic bacteria, biofilm bacteria are 10–1,000 times more resistant to antibiotics (Stewart and Costerton, 2001). Nearly all marine structures in seawater are colonized with biofilm-forming bacteria. Biofoulers colonize ship hulls, underwater pipelines, and marinas, causing a substantial economic loss in marine operations and environmental problems such as the introduction of invasive species (Hellio and Yebra, 2009). For example, ships need additional power due to the extra burden from biofouler attachment, leading to high fuel consumption and heavy engine stress (Bixler and Bhushan, 2012).

Various antifouling technologies have been developed to prevent biofouling, including the addition of antifouling agents such as copper pyrithione, chlorothalonil, zinc pyrithione, and SeaNine 211 to marine paints (Qian et al., 2013). Besides these, antifouling paint containing biocide tributyltin (TBT) is highly efficient in preventing the settlement and growth of biofoulers but it is toxic toward non-target organisms and persistent in marine environments; hence, its application on ships has been banned by the International Maritime Organization (IMO) (Champ, 2000). Thus, there is an urgent need to develop non-toxic, cost-effective, and environmentally friendly antifoulants.

In recent years, antifouling compounds derived from various natural sources have been discovered and considered to be degradable and low toxic to the marine environment (Qi and Ma, 2017). Especially, bacterial sources of bioactive compounds are preferable as they can be reproduced and scaled up for ensuring product supply for commercialization (Heidarian et al., 2019). It is undeniable that natural products isolated from bacterial fermentation are vast resources for the exploitation of antifouling compounds. For example, butenolide isolated from the marine bacterium *Streptomyces albidoflavus* prevents the settlement of dominant fouling organisms (barnacles, tubeworms, and bryozoans) with a low toxic effect (Xu et al., 2010); 3,3-Diindolylmethane isolated from *Pseudovibrio denitrificans* exhibits antifouling activities against barnacles and bryozoans with equivalent field performance to that of the commercial antifouling agent SeaNine 211 (Wang et al., 2015). Previously reported natural products with antifouling activity also include fatty acids, lactones, terpenes, steroids, benzenoids, phenyl ethers, polyketides, alkaloids, nucleosides, and peptides (Wang et al., 2017). However, it is difficult to develop marine natural product-based antifouling coatings because of the quick release of antifouling compounds, the complicated procedure for chemical synthesis, and the low yields of the antifoulants (Sisson et al., 2013). To solve problems related to compound release control, the self-polishing copolymer, which uses silyl acrylate as a carrier of antifoulants, generates a self-renewing surface through hydrolysis and degradation and thus, controls antifouling compound release (Bressy et al., 2010; Xie et al., 2019). To solve the compound supply issue, chemical synthesis or biosynthesis of target antifouling compounds are common practices. Yet, there have been no studies that address these challenges in a holistic (systematic) way.

Albofungin and its derivatives were identified as secondary metabolites from various *Streptomyces* species, and they belong to polycyclic isoquinolone–xanthone family. Albofungin and chloroalbofungin have been crystallized and their structures were determined by single-crystal X-ray diffraction (Ye et al., 2020). Our previous study has reported remarkable antibacterial activities against “ESKAPE pathogens” and antitumor activities of albofungin and its derivatives isolated from the bacterium *S. chrestomyceticus* BCC 24770 (She et al., 2021). In the present study, the antifouling activities of four albofungins were firstly evaluated. Since the albofungin biosynthesis pathways and their regulatory genes in *S. chrestomyceticus* have been already known (She et al., 2021), it is a natural extension of our current work to develop high-yield engineered strains to improve albofungin production through manipulation of its biosynthesis gene cluster in *S. chrestomyceticus*. Based on their structure-activity relationship and production analyses, the albofungin-based coatings were further prepared and evaluated in marine environments.

## Materials and Methods

### General Experimental Procedures

The strains and plasmids are listed in Table 1. *Staphylococcus* sp. Z01 and *Micrococcus* sp. Z02 were isolated from marine biofilm grown on Petri dishes (Corning Inc., New York, United States) in a subtidal zone as described in Wang et al. (2020). The 16S rRNA genes amplicon was performed using 8F/1492R primers, followed by Sanger sequencing in BGI (Beijing, China). BLAST searches on the NCBI 16S ribosomal RNA sequences database and EzBioCloud database was performed on the obtained sequences to identify the taxonomy of the isolates. The 16S rRNA gene sequence of *Micrococcus* sp. has 94.7% of similarity *to Micrococcus yunnanensis*, and that of *Staphylococcus* sp. has 96.2% of similarity *to Staphylococcus warneri*.

**TABLE 1:** Bacteria strains and plasmids.

Strain or plasmid	Characteristics	References
*Escherichia coli* TOP10	Cloning host	O’Sullivan et al., 2001
*E. coli* ET12567/pUZ8002	*Streptomyces* conjugation	Guan and Pettis, 2009
MCCC 1A01390 *Pseudomonas pachastrellae*	Biofilm formation strain	Marine Culture Collection of China (MCCC)
MCCC 1A04899 *Sulfitobacter pontiacus*	Biofilm formation strain	Marine Culture Collection of China (MCCC)
MCCC 1A11723 *Psychrobacter nivimaris*	Biofilm formation strain	Purchased from the Marine Culture Collection of China (MCCC)
*Micrococcus* sp. Z02	Biofilm formation strain	This study
*Staphylococcus aureus* B04	Biofilm formation strain	Culture collection of our laboratory
*Staphylococcus* sp. Z01	Biofilm formation strain	This study
*Streptomyces* strains		
*Streptomyces chrestomyceticus* BCC 24770	Parental strain to produce albofungin	
24770/pPWW-*alb45*	Overexpression strain	This study
24770/pPWW-*alb22*	Overexpression strain	This study
**Plasmids**		
pPWW50a	Cloning and expression vector	Malit et al., 2021
pPWW-*alb45*	pPWW50a with *alb45* expression under the control of the strong constitutive promoter ermE*p	This study
pPWW-*alb22*	pPWW50a with *alb22* expression under the control of the strong constitutive promoter ermE*p	This study

Albofungins were isolated and purified as previously described (She et al., 2021). The purity of tested compounds was confirmed by high-performance liquid chromatography (95% purity, HPLC, Waters 2695, Milford, MA, United States), and their structures were determined by the Bruker NMR spectrometers (Bruker, Billerica, MA, United States) and X-ray crystallography as previously described (Ye et al., 2020; She et al., 2021).

### Assessment of Biofilm Formation by MTT Assay

The ability to form static biofilms of the 6 marine bacteria was tested. Marine bacteria were cultured overnight in marine broth at 22°C (*Pseudomonas pachastrellae* MCCC 1A01390, *Sulfitobacter pontiacus* MCCC 1A04899, and *Psychrobacter nivimaris* MCCC 1A11723) or 30°C (*Staphylococcus aureus* B04, *Staphylococcus* sp. Z01, and *Micrococcus* sp. Z02) with 220 rpm agitation and then diluted into approximately 10^7^CFU mL^–1^ in marine broth supplemented with 1% of glucose. Afterward, 200 μL of the diluted solution were added to each well of a 96-well plate (Corning Inc., New York, United States) and was then incubated at 22 or 30°C for 24 h. The culture medium, planktonic cells and loosely adhered bacteria were removed by dual washing with a phosphate-buffered saline (PBS) buffer, and the firmly attached bacteria were incubated with 20 μL of MTT (5 mg mL^–1^) at 37°C for 3 h. The supernatant was discarded, and formazan was dissolved in 150 μL of 100% DMSO (Sigma-Aldrich, St. Louis, United States). Absorbance was measured using the Multiskan™ FC microplate photometer (Thermo Fisher Scientific, Waltham, United States) under 570 nm, and *Staphylococcus aureus* ATCC 43300 was used as a positive control. All the experiments were performed in triplicate.

### Antibacterial Assay and Antibiofilm Assay

The antibacterial activities of albofungins against the marine bacteria were tested following specific protocols (She et al., 2020). Marine broth was used as the test medium for the marine bacteria. The plate was kept at 22 or 30°C overnight. The minimum inhibitory concentration (MIC) which means the lowest concentration that the drug prevents the visible growth of bacteria was used to evaluate antibacterial assay.

The marine bacteria which have successfully formed biofilms were further used for antibiofilm assays. Different concentrations of albofungin compounds were added to each well. Biofilm formation was assessed through the MTT assay as previously described. The minimum biofilm inhibitory concentration (MBIC_90_) which refers to the lowest concentration of a drug to effectively inhibit 90% of the biofilm formation was calculated. Data were analyzed using one-way ANOVA to detect significant differences and standard deviation (SD) was calculated using GraphPad Prism 9. All the assays were performed in triplicate.

### Collection, Culturing, and Anti-larval Settlement Bioassay of Barnacle *Amphibalanus amphitrite* Larvae and Bryozoan *Bugula neritina* Larvae for Anti-larval Settlement Bioassay

*A. amphitrite* adults were collected from Pak Sha Wan Pier, Hong Kong (22°38′N, 114°28′E) and after keeping in dark for 24 h, a light source (LED lamp, 1,500 lumens) was used to stimulate the larval release. Within 1 h, the larvae were collected and cultured in 0.22 μm filtered seawater with a daily diet of *Chaetoceros gracilis* Schutt at 1 × 10^6^ cells mL^–1^ until their growth into cyprids, which were used for the anti-larval settlement bioassay. The bryozoan *B. neritina* adults were collected from a fish farm in Pak Shek Kok, Hong Kong (22°43′N, 114°20′E) and kept in flow-through seawater for no more than 7 days before use. The larvae were released within 30 min before the bioassay as described (Xu et al., 2010).

The bioassay was conducted in a 24-well polystyrene tissue culture plate with 15–20 larvae in each well. 0.1% of DMSO in filtered seawater (FSW), and 0.625, 1.25, 2.5, 5, 10, 20, and 40 μg mL^–1^ albofungin concentrations were tested in triplicate. In each well, 1 mL of FSW containing 15–20 larvae and 1 mL of albofungin solution of different concentrations. The plate was kept for 48 h at 25°C (bryozoan *B. neritina* larvae were kept for 3 h). The wells with 0.1% of DMSO in FSW served as a negative control, and butenolide was used as a positive control. The number of attached, swimming, and dead larvae were counted under an Olympus optical microscope (Olympus Corporation, Tokyo, Japan). The settlement rate was calculated as the ratio of settled larvae to the total number of larvae in each well, and the death rate was calculated as the ratio of dead or missing larvae to the total number of larvae in each well. Half maximal effective concentration (EC_50_) and half lethal concentration (LC_50_) were determined for each compound, and the ratio of LC_50_/EC_50_ was used to evaluate the toxicity of the antifoulant. Experiments were performed in three independent batches of larvae. Data were analyzed by one-way ANOVA to detect significant differences in the larval settlement, and SD was calculated by GraphPad Prism 9.

### Construction of Activator Overexpressed *Streptomyces chrestomyceticus* and Analysis of Albofungin Production

The overexpression plasmids were constructed as follows. Gene sequences were obtained by PCR using the primers listed in Supplementary Table 1 with the genomic DNA of *S. chrestomyceticus* as the template. Each PCR amplicon was ligated into the linear vector pPWW50a digested with *Nde*I and *Spe*I under the control of strong constitutive promoter ermE*p. All the constructed plasmids were confirmed by DNA sequencing, introduced into *E. coli* ET12567/pUZ8002, and conjugated to *S. chrestomyceticus* BCC 24770 following a previous protocol (Gust et al., 2003). The conjugants were collected and grown on a selective plate containing apramycin (25 μg mL^–1^) and nalidixic acid (25 μg mL^–1^). After 3 days, total DNA was extracted using Chelex 100 resin (Bio-Rad, Hercules, United States) and PCR amplification for the positive conjugants 24770/pPWW-*alb22* and 24770/pPWW-*alb45* (Supplementary Figure 15, using primers pPWW50a-check-F and pPWW50a-check-R). BCC24770 with the empty plasmid was used as a negative control (24770/pPWW). The positive conjugants of *S. chrestomyceticus* 24770/pPWW-*alb22* and *S. chrestomyceticus* 24770/pPWW-*alb45* and parental strain *S. chrestomyceticus* 24770/pPWW were inoculated into seed medium (4 g L^–1^ glucose, 4 g L^–1^ yeast extract, 10 g L^–1^ malt extract, and pH being adjusted to 7.0–7.4) with apramycin (50 μg mL^–1^) and grown for 2 days. Afterward, 1% of the preculture was added into the fermentation medium (4 g L^–1^ glucose, 4 g L^–1^ yeast extract, 10 g L^–1^ malt extract, and pH being adjusted to 7.0–7.4) with or without apramycin (50 μg mL^–1^) and grown for 9 days in the 250 mL shaking flasks (Corning Inc., New York, United States) at 220 rpm. The fermentation products were further analyzed by HPLC. The production of albofungin was calculated according to the standard curve (see Supplementary Figure 14).

*S. chrestomyceticus* samples were collected and washed using autoclaved water at the end of third day of fermentation and immediately frozen at −80°C for RNA extraction. The RNA samples were prepared using Trizol Reagent (Invitrogen, Waltham, United States) following the manufacturer’s instructions. A HiScript III All-in-one RT SuperMix Perfect for qRT-PCR kit (Vazyme, Nanjing, China) was used to remove genomic DNA and synthesize cDNA. The qRT-PCR analysis was performed on Roche Diagnostics LightCycler 480 Instrument II Real-time PCR System using LightCycler^®^ 480 SYBR Green I Master (Roche, Basel, Switzerland). The primers used are listed in Supplementary Table 1. The *GAPDH* gene (glyceraldehyde-3-phosphate dehydrogenase) was used as an internal control, and the relative expression levels of *alb22* and *alb45* were normalized to *GAPDH.* Each transcript was performed in triplicate and repeated for three independent biological replicates in qRT-PCR. The relative fold changes in the expression level of each gene were calculated using the 2^–ΔΔ*CT*^ method (Livak and Schmittgen, 2001). The *p*-value is computed using Student’s *t*-test.

### Albofungin-Based Coating Preparation and Release Rate Measurement

Around 1 g of albofungin compound was isolated, purified, and analyzed using HPLC to guarantee over 95% purity. Albofungin-based hydrolyzable and degradable copolymer coating was prepared as follows: methyl methacrylate (MMA) and tributylsilyl methacrylate (TBSM) copolymer (PMSM0) was synthesized via radical ring-opening polymerization (Zhou et al., 2015). For 5 wt% of albofungin-based coating, 0.95 g of PMSM0 and 0.05 g of albofungin were dissolved in xylene and tetrahydrofuran (v:v = 1:2) and mixed vigorously at room temperature. The solution was then coated on the PVC panels (4 × 7 cm^2^) and dried at room temperature for 7 days (Ma et al., 2017). Other coatings with different albofungin concentrations (10 and 15 wt%) were prepared using the same procedure. The three concentrations of albofungin-based coatings were optimized based on our previous findings (Wang et al., 2015; Ma et al., 2017). The coating with PMSM0 only was used as a positive control. Each concentration was prepared for three biological replicates. The field test was conducted from March to April 2021 in a fish farm at Yung Shue O, Hong Kong (22°24′N, 114°21′E), which was heavily fouled all year round. The PVC panel was submerged into seawater at a depth of 0.5 m for 2 months, retrieved, washed with seawater, and photographed. The area covered by biofoulers was calculated by Image J (Fiji-2.2.0) (Schindelin et al., 2012). One-way ANOVA was used to compare the albofungin-coated panels and the control panels. The release rate of albofungin under static conditions was determined by measuring the compound concentration with HPLC. Albofungin-based coatings were applied onto a PVC panel (20 × 75 mm^2^) and submerged in artificial seawater (ASW, NaCl 24.53 g, MgCl_2_⋅6H_2_O 11.09 g, Na_2_SO_4_ 4.90 g, CaCl_2_ 1.16 g, KCl 6.95 g, NaHCO_3_ 0.201 g, KBr 0.101 g, H_3_BO_3_ 0.027 g, SrCl_2_⋅6H_2_O 0.042 g, NaF 0.003 g per liter of water). After 7 days, the panel was transferred to an individual container with 100 mL of ASW. After 24 h of immersion, 10 mL of the seawater was taken out of the container and extracted with the same volume of ethyl acetate three times. After drying under the SpeedVac vacuum concentrators, the extracts were dissolved in 100 μL of methanol and then subjected to HPLC using a reversed-phase system (Waters 2695) with a Phenomenex Luna C18 column connected to a UV detector at 300 nm. The unique UV absorption of albofungin and retention time were determined, with the amount being calculated from the established standard curves using peak areas plotted against known standard quantities. The logarithmic function was used in curve fitting according to the average of the value.

### Nucleic Acid Extractions, 16S rRNA Amplicon Sequencing and Analysis

The field test for biofilm formation was performed in Pak Sha Wan Pier, Hong Kong (22°38′N, 114°28′E). Glass slides with albofungin/copolymer coatings were submerged at a depth of 0.5 m from sea surface for 12 days and immediately transported into the laboratory for biofilm extraction. The biofilm was scraped using a sterilized cotton swab and was collected in TE buffer (10 mM Tris-Cl; 0.5 mM EDTA). The samples were centrifuged at 4,000 rpm for 5 min and the supernatant was discarded. Bacteria genomic DNA extraction kit (TIANGEN, Beijing, China) was used to extract the genomic DNA, and the quality was confirmed through BioDrop (Biochrom Ltd., Cambridge, United Kingdom). The 16S rRNA amplicon sequencing of the extracted genomic DNA was performed using an Illumina paired-end platform to generate 250 bp paired-end raw reads (Raw PE) in Novogene (Beijing, China). Sequence data of six samples were subjected to quality control and analyzed using the microbial ecology community software program Mothur (Schloss et al., 2009). Low-quality reads (average quality score < 25) and reads with incorrect length (no shorter than 400 bp and no longer than 430 bp), any ambiguous base, and homopolymers longer than 8 bp were removed. Chimeric sequences were identified and removed by Chimera.uchime in Mothur package. The remaining high-quality sequences were then clustered into the operational taxonomic unit (OTUs) at 97% similarity. Singletons and doubletons were removed before downstream analysis. Taxonomic annotation was performed using Classify. OTU in Mothur with Silver.132 database.

## Results

### Structures of Albofungins

Four compounds (**1**–**4**) in total were isolated: that are, albofungin (**1**), its dimethoxy product, albofungin A (**2**), its monochlorinated derivative, chloroalbofungin (**3**), and its deaminated derivative, chrestoxanthone A (**4**) (Figure 1A). All of them were extracted at a high amount from the cultures of *S. chrestomyceticus* BCC 24770 (Bunyapaiboonsri et al., 2016; She et al., 2021). The structure elucidation of albofungins (**1**–**4**) was carried out as described in Supplementary Figures 1–13.

**FIGURE 1:**
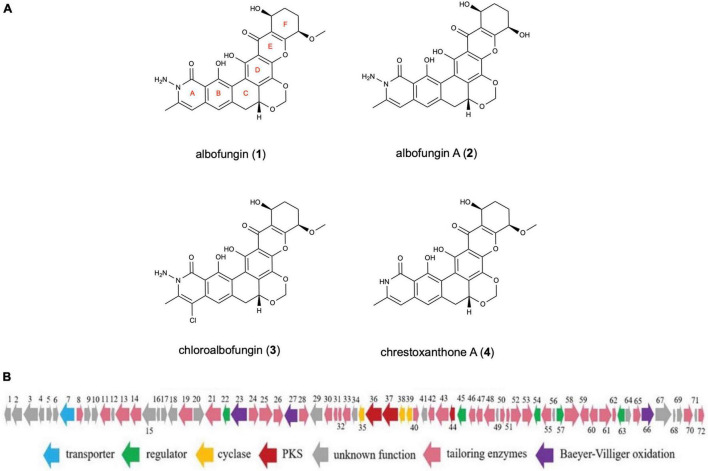
**(A)** Chemical structures of albofungins (**1**–**4**) isolated from *Streptomyces chrestomyceticus* BCC 24770. **(B)** Genetic organization of albofungin biosynthesis gene cluster (She et al., 2021).

### Antibiofilm Activities of Albofungins Against Microfouling Bacteria

**FIGURE 2:**
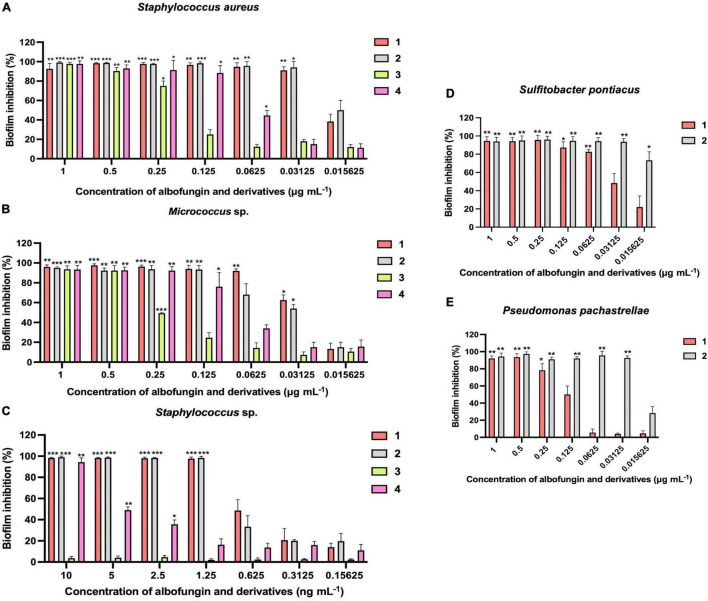
Inhibition of biofilm formation of *Staphylococcus aureus* **(A)** *Micrococcus* sp. **(B)** *Staphylococcus* sp. **(C)** *Sulfitobacter pontiacus* **(D)** *Pseudomonas pachastrellae* **(E)** by albofungins (**1**–**4**). Error bars represent SD, *n* = 9 wells from 3 batches of microbial cultures. Significant differences were analyzed by one-way ANOVA compared with the control biofilm, *p < 0.05, **p < 0.01, and ***p < 0.001.

The antibiofilm activity of albofungins (**1**–**4**) was evaluated using 6 species of marine bacteria, either isolated from subtidal marine biofilms or described as dominant primary colonizers of submerged surfaces, including *Staphylococcus aureus*, *Micrococcus* sp., *Staphylococcus* sp., *Sulfitobacter pontiacus*, *Pseudomonas pachastrellae*, and *Psychrobacter nivimaris*. The results showed that albofungins (**1**–**4**) strongly prevented the biofilm formation of all selected strains (Table 1). The MBIC_90_ values of compounds **1**–**4** against the biofilm formation of Gram-positive bacteria were at a low micromolar range (Table 2). More specifically, biofilm formation of *S. aureus*, *Micrococcus* sp., and *Staphylococcus* sp. was almost completely inhibited (>90%) by compounds **1**–**4** at concentrations ranging from 0.03 to 0.5 μg mL^–1^, 0.06 to 0.5 μg mL^–1^, and 1.25 ng mL^–1^ to 0.2 μg mL^–1^, respectively (Figure 2). As for Gram-negative bacterial strains, compounds **1** and **2** showed strong antibiofilm activities against *S. pontiacus*, and *P. pachastrellae* with MBIC_90_ ranging from 0.02 to 0.50 μg mL^–1^, whereas compounds **3** and **4** displayed only moderate activities against these bacteria at a concentration of 10–20 μg mL^–1^ (Table 3). All of the compounds, however, showed no apparent biofilm inhibition effects against *P. nivimaris* at concentrations of less than 20 μg mL^–1^. Additionally, compounds **1**–**4** exhibited antibacterial activities against both Gram-positive bacteria with MIC ranging from 0.8 to 50 ng mL^–1^ and Gram-negative bacteria with MIC ranging from 0.008 to 20 μg mL^–1^ (Supplementary Figures 18, 19). These results implied that the biofilm inhibition of albofungins is possibly owing to the inhibition of bacterial growth. Consistent with the antibacterial results, compounds **1** and **2** that contain the hydrazine group but without a chlorinated ring A, exhibited stronger antibiofilm activities than compounds **3** and **4**. According to the preliminary structure-activity relationship analysis, the existence of a hydroxyl group in ring F in compound **2**, instead of a methoxy group in comparison with compound **1**, allows for higher antibiofilm activities against Gram-negative bacteria.

**TABLE 2:** Biofilm inhibition assay of albofungins (**1**–**4**) against gram-positive marine bacteria.

Gram-positive bacteria	*Micrococcus* sp. Z02	*Staphylococcus aureus* B04	*Staphylococcus* sp. Z01
**Antibiofilm MBIC_90_ (ng mL^–1^)**			
Albofungin **(1)**	31.25–62.50	15.63–31.25	0.63–1.25
Albofungin A **(2)**	62.5–125	15.63–31.25	0.63–1.25
Chloroalbofungin **(3)**	250–500	250–500	100–200
Chrestoxanthone A **(4)**	125–250	62.5–125	5–10

*The results are represented as MBIC_90_ in ng mL^–1^.*

**TABLE 3:** Biofilm inhibition assay of albofungins (**1**–**4**) against gram-negative marine bacteria.

Gram-negative bacteria	MCCC 1A04899 *Sulfitobacter pontiacus*	MCCC 1A01390 *Pseudomonas pachastrellae*	MCCC 1A11723 *Psychrobacter nivimaris*
**Antibiofilm MBIC_90_ (μg mL^–1^)**			
Albofungin **(1)**	0.13–0.25	0.25–0.50	10–20
Albofungin A **(2)**	0.02–0.03	0.02–0.03	>20
Chloroalbofungin **(3)**	10–20	>20	>20
Chrestoxanthone A **(4)**	10–20	>20	>20

*The results are represented as MBIC_90_ in μg mL^–1^.*

### Antifouling Activity of Albofungins Against the Barnacle *Amphibalanus amphitrite* and Bryozoan *Bugula neritina* Larvae

**FIGURE 3:**
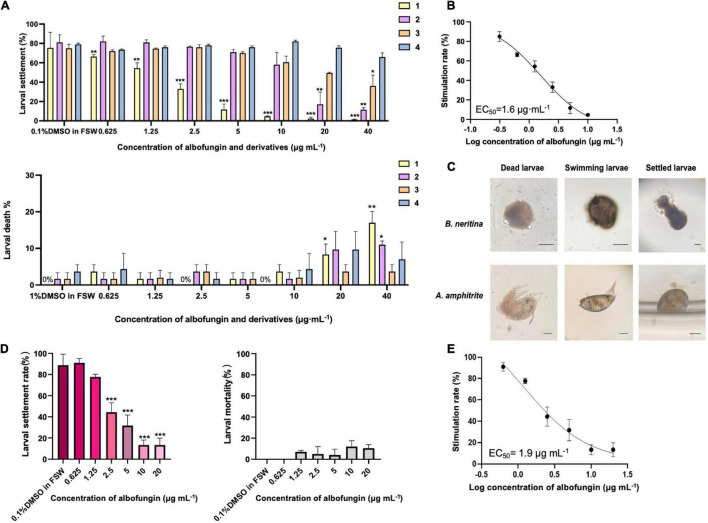
Antifouling activity of albofungins (**1**–**4**) against barnacle *Amphibalanus amphitrite* and bryozoan *Bugula neritina*. **(A)** Larval settlement rate and larval mortality rate of *A. amphitrite* under the treatment of albofungins (**1**–**4**) with concentrations of 0.625–40 μg mL^–1^ for 48 h. Error bars represent SD, *n* = 9 wells from 3 batches of larval cultures. Significant differences were analyzed by one-way ANOVA, *p < 0.05, **p < 0.01, and ***p < 0.001. **(B)** Stimulation rate curve of albofungin (**1**) treatment on *A. amphitrite* larvae. Error bars represent SD, *n* = 9 wells from 3 batches of larval cultures. **(C)** Different conditions of *A. amphitrite* and *B. neritina* larvae during the bioassay. Scale bars = 100 μm. **(D)** Larval settlement rate and larval mortality rate of *B. neritina* under the treatment of albofungin (**1**) with concentrations of 0.625–20 μg mL^–1^ for 3 h. Error bars represent SD, *n* = 9 wells from 3 batches of larval cultures. Significant differences were analyzed by one-way ANOVA, *p < 0.05, **p < 0.01, and ***p < 0.001. **(E)** Stimulation rate curve of albofungin (**1**) treatment on *B. neritina* larvae. Error bars represent SD, *n* = 9 wells from 3 batches of larval cultures.

Then the anti-macrofouling activities of albofungins (**1**–**4**) against the larval settlement of the barnacle *A. amphitrite* and bryozoan *B. neritina* were evaluated. The results showed that the settlement rate of *A. amphitrite* was significantly lower in the treatments of compounds **1** and **2** than that of the control group (0.1% DMSO in FSW) after 48 h of incubation, whereas compounds **3** and **4** did not cause significant difference from the FSW control group (Figures 3A,C). The larval settlement rate of *A. amphitrite* in 2.5 μg mL^–1^ of compound **1** treatment was 33% (± 5.3%), and in 20 μg mL^–1^ of compound **2** treatment was 17% (± 12.7%), both of which were significantly lower than that in the FSW control group. With respect to *A. amphitrite*, compound **1** exhibited a strong inhibitory effect with an EC_50_ of 1.6 μg mL^–1^, and compound **2** showed a moderate inhibitory effect with an EC_50_ of 12.2 μg mL^–1^ (Figure 3B and Supplementary Table 2). Among these tested compounds, compound **1** showed equivalent antifouling activity against the larval settlement of *A. amphitrite* to that of butenolide, which is a highly potent antifouling compound according to the EC_50_ value (Supplementary Table 2). In particular, albofungins up to 40 μg mL^–1^ had very low lethal effects (Figure 3A). Meanwhile, the antifouling activity of compound **1** against the bryozoan *B. neritina* larvae was concentration-dependent and showed low lethal effects at the highest concentration tested as well in the present study (Figures 3D,E). Treatment with 2.5 μg mL^–1^ of compound **1** had 44% (± 9.0%) of larval settlement of *B. neritina*, which was significantly lower than that in the FSW control group (Figure 3D).

### Overexpression of the Candidate Activator Genes

**FIGURE 4:**
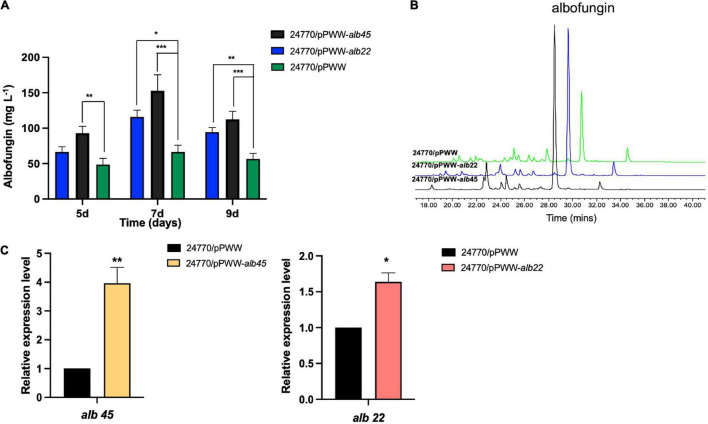
Effects of overexpressed regulators on albofungin production. **(A)** Albofungin production (mg L^–1^) by 24770/pPWW (parental strain), 24770/pPWW-*alb22* and 24770/pPWW-*alb45* overexpression strains. Error bars represent SD, *n* = 3 independent cultures. Significant differences were analyzed by one-way ANOVA, *p < 0.05, **p < 0.01, and ***p < 0.001. **(B)** HPLC analysis (300 nm) of the crude extracts of 24770/pPWW, 24770/pPWW-*alb45* and 24770/pPWW-*alb22* at 7 days. **(C)** Relative expression level of *alb45* and *alb22* in 24770/pPWW, 24770/pPWW-*alb45* and 24770/pPWW-*alb22* strains. GAPDH was used as a reference gene, and 24770/pPWW served as a control. Error bars represent SD, *n* = 3 independent cultures. Significant differences were analyzed by Student’s *t*-test, *p < 0.05, **p < 0.01, and ***p < 0.001.

To improve the production of albofungin, transcriptional regulators alb22 and alb45 were overexpressed separately in the *S. chrestomyceticus* BCC 24770. The qRT-PCR analysis revealed that the transcription levels of the two regulatory genes were increased by 0.6- and 3.0-fold in the *alb22* and the *alb45* overexpression strains compared with those in the parental strain (Figure 4C). This finding further supported the successful overexpression of these two regulatory genes. Meanwhile, the fermentation results of overexpression strains and parental strain were analyzed using HPLC and the results showed that the overexpression of *alb22* and *alb45* improved albofungin production by 37 and 91% in comparison with the parental strain after 5 days. Noticeably, after 7 days of cultivation, 24770/pPWW-*alb22* strain and 24770/pPWW-*alb45* strain produced 115 ± 9.4 and 153 ± 22.7 mg L^–1^ albofungin in parallel fermentations, which were 0.7- and 1.3-fold higher yields than the parental strain (Figures 4A,B), respectively. Because albofungin is yellow powder, its yield can be indicated by the color of the crude extract. Clearly, the color of crude extract from 24770/pPWW-*alb22* and 24770/pPWW-*alb45* dissolved in methanol was darker than that of the parental strain (Supplementary Figure 16). Additionally, the overexpression of regulatory genes showed no significant influence on the growth of *S. chrestomyceticus* (Supplementary Figure 17). These findings suggested 24770/pPWW-*alb45* as the preferrable genetically modified strain for albofungin production.

### Antifouling Efficacy of Albofungin and Degradable Copolymer Coatings in Marine Field Test and Release Rate Determination

**FIGURE 5:**
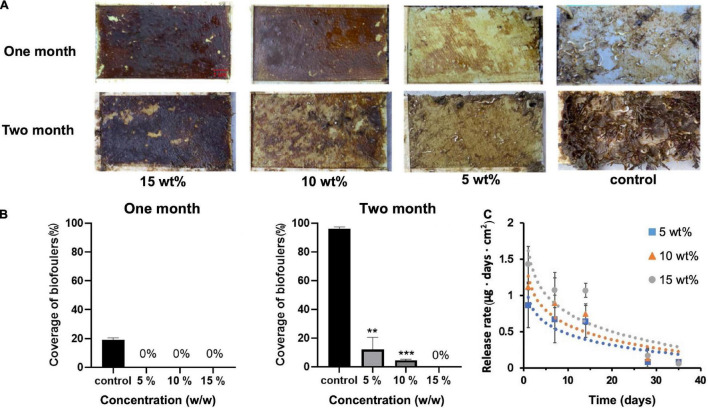
Antifouling effect of albofungin-based copolymer coating in the field test after 2 months. **(A)** The copolymer was coated with 5, 10, and 15 wt% albofungin on PVC panels. Control was coated with copolymer only. **(B)** Percentage of coverage of biofoulers after 1 and 2 months. Error bars represent SD (*n* = 3). Significant differences were analyzed by one-way ANOVA, **p < 0.01, and ***p < 0.001. **(C)** Time-dependent release rate of albofungin into artificial seawater. Error bars represent SD (*n* = 3). Significant differences were analyzed by one-way ANOVA and no significant differences were found between 5, 10, and 15 wt% albofungin-based coating groups on the same day.

Albofungin (**1**) showed remarkable anti-microfouling and anti-macrofouling activities with low toxicity toward the target organisms, meanwhile, large-scale fermentation of albofungin high-yield strain could easily provide a gram-scale amount of the compound under laboratory conditions at a low cost. Thus, the antifouling efficacy of albofungin was assessed in a field study. 1 g of pure albofungin (**1**) was obtained from 7 L of *S. chrestomyceticus* 24770/pPWW*-alb45* bacterial culture and then incorporated into different antifouling coatings. These coatings were then applied onto PVC panels that were submerged in marine environments for 60 days. Noticeably, almost no macrofoulers settled onto the surfaces of the albofungin-coated PVC panels in all three concentrations (5, 10, and 15 wt%) after 1 month (Figure 5A), whereas the surface of the negative control group was fouled by the bryozoan *B. neritina* and the polychaete *H. elegans*, which is the most widespread tubebuilding worm in tropical and subtropic regions. After 2 months of submerging in the sea, 96% of the PVC panel surface area was covered by macrofoulers in the control group, whereas the regions fouled in albofungin-coated groups were significantly smaller (Figures 5A,B).

The release rate of albofungin from coatings at different concentrations into artificial seawater was measured within 35 days. Overall, the release rate was low during the whole observation period and was positively correlated with the albofungin concentration. The highest release rate of 15, 10, and 5 wt% of albofungin reached 1.4, 1.1, and 0.86 μg day^–1^ cm^–2^ on the first day, respectively, and decreased to approximately 0.08 μg day^–1^ cm^–2^ in a time-dependent manner (Figure 5C).

### Changes in Microbial Community Structure Caused by Albofungin and Degradable Copolymer Coatings

**FIGURE 6:**
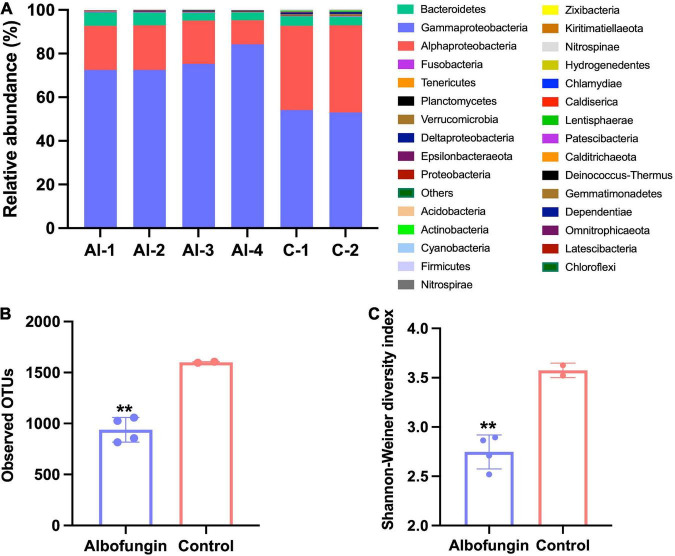
Microbial community structure alteration by albofungin-based copolymer coatings based on 16S rRNA amplicon analysis. **(A)** Relative abundance of the major bacterial phylum of biofilms developed in 5 wt% albofungin-based copolymer coatings and control with only copolymer coatings (Proteobacteria are further classified into the classes). **(B)** Observed OTUs of albofungin-based copolymer coatings and control with only copolymer coatings. Error bars represent SD. Significant differences were analyzed by Student’s *t*-test, **p < 0.01. **(C)** Shannon–Weiner diversity index of albofungin-based copolymer coatings and control with only copolymer coatings (al-1, al-2, al-3, al-4, and control-1, control-2). Error bars represent SD. Significant differences were analyzed by Student’s *t*-test, *p < 0.05, **p < 0.01, and ***p < 0.001.

Biofilm development on albofungin and degradable copolymer coatings in the field was further examined. During 12 days of observations, the biofilm quickly grew on the panel surfaces in the control group and consisted of diverse microorganisms whereas the diversity of microorganisms on the panel surface covered with 5 wt% of albofungin-based coating reduced significantly. A total of 1,687,617 sequencing reads were analyzed from 6 biofilm samples and microbes in those biofilms were classified into 31 phyla. The 12-day biofilm, which was hereby referred to as “old biofilms” (12–20 days biofilms) (Chung et al., 2010), was dominated mainly by Proteobacteria (Gammaproteobacteria, Alphaproteobacteria, Deltaproteobacteria), Bacteroidetes, Verrucomicrobia, and Actinobacteria (Figure 6A). Alpha diversity was used to indicate microbial community diversity in the albofungin-based coating group and control group. The results of the Shannon-Weiner diversity index and observed OTUs were significantly reduced in the albofungin-based coating group, indicating an altered microbial community structure (Figures 6B,C). The richness and diversity of microbial communities were also lower than those of the control group.

## Discussion

The albofungin and its derivatives isolated from *S. chrestomyceticus* in this study are belong to polycyclic xanthones. In the family of polyketides, polycyclic xanthones are prominent members, but little research has been done on their antifouling activity (Wang et al., 2017). As previously reported, albofungins showed various biological activities such as potential antibiotics against “ESKAPE pathogens,” antifungal activities against pathogenic fungi, and antitumor activities against different cancer cell lines (Bunyapaiboonsri et al., 2016; She et al., 2021). The strong antibiotic effect of albofungins against Gram-positive bacteria was linked to the presence of a hydrazine group (She et al., 2021). In addition, albofungin A inhibited cancer cell proliferation by inducing cellular apoptosis (She et al., 2021). Owing to their prominent and diverse bioactivities, albofungins might have great potential in biofilm control and thus anti-microfouling activities.

In this study, the antifouling activities of albofungins were firstly evaluated. The antibiofilm activities results suggested compounds **1** and **2** contained the hydrazine group had better activities against both gram-positive and gram-negative bacteria. Actually, a similar result was reported in the assessment of bioactivity of hydrazone derivatives, where hydrazide-hydrazones were found to be less active than hydrazides due to the blockage of the –NH_2_ group (Rollas and Küçükgüzel, 2007). Given that these compounds displayed potent antibiofilm activity, they could be used in preventing undesired microfouling in natural environments. Similar to what was observed with antibiofilm testing, the hydrazine group without chlorination on ring A found in albofungin could cause anti-larval settlement activity. As a compound with EC_50_ < 5 μg mL^–1^ and LC_50_/EC_50_ > 50 can be viewed as a non-toxic and high potential antifouling compound (Qian et al., 2009), compound **1** with a high LC_50_/EC_50_ ratio shall be regarded as a low-toxic antifoulant with good antifouling activity. Albofungin has been reported as an inhibitor of bacterial transglycosylase of penicillin-binding protein (Wu et al., 2018), therefore, we proposed albofungins inhibit biofilm formation by cleaving peptidoglycan, which is present in the cell wall of tested bacteria (Roy et al., 2018). The anti-larval settlement assay showed low cytotoxicity against the larvae after 24 h, and according to our observations, the larvae tended to swim inactively. As previously reported that the energy metabolism of larvae is highly needed during the settlement process (Zhang et al., 2010), so we hypothesized that the albofungins blocked the energy metabolism of the larvae during their settlement process, which needs further investigation.

Given that the total synthesis of albofungin remains challenging, albofungin high-producing strains are needed to satisfy the demand for field test and further application of this great potential compound. Heterologous expression and genetic manipulation are important tools for constructing high-yield strains (Li et al., 2015; Bian et al., 2017). Our previous study revealed that a bacterial artificial chromosome plasmid containing an albofungin biosynthesis gene cluster (72 kb) was successfully introduced into *Streptomyces coelicolor* to yield the albofungin (Figure 1B). However, the production of albofungin in *S. coelicolor* M1146 4L19 was lower than that in *S. chrestomyceticus* strain. A similar phenomenon has also been reported in other studies. The heterologous expression of potential anticancer drugs epothilones in *S. colicolor* leads to a low yield, possibly owing to its cytotoxic effect toward heterologous host (Julien and Shah, 2002). It implied that albofungin could inhibit the growth of spores from *S. coelicolor* and *S. chrestomyceticus*. And the results indicated that albofungin showed stronger inhibitory effects towards the heterologous host than the wild-type strain. Thus, genetic manipulation in wild-type *S. chrestomyceticus* was the ideal approach to improve the yield of albofungin.

It is known that the positive regulators activate the transcription of targeted biosynthesis gene clusters, and overexpressing the positive regulators can efficiently optimize the production of important secondary metabolites (Baral et al., 2018; Wei et al., 2018; Xia et al., 2020). *Alb22* and *alb45* encoded for transcriptional enhancer A (TenA) family regulator and *Streptomyces* antibiotic regulatory protein (SARP) family regulator, which were identified as positive regulators for albofungin biosynthesis (She et al., 2021). They are located closer to the polyketide synthase genes than other regulators. The TenA family transcriptional regulator was reported to stimulate the production of extracellular proteases in *Bacillus subtilis* and exhibits an enzymatic function in thiamine metabolism in *Pyrococcus furiosus* (Benach et al., 2005; Lee et al., 2005). Moreover, a crystal structure of TenA regulator revealed that it activates gene expression indirectly (Benach et al., 2005), which may imply indirect transcriptional mechanisms of *alb22* in the albofungin biosynthesis gene cluster. However, further study is required to confirm this notion. Meanwhile, many SARPs positively regulate secondary metabolites in *Streptomyces* (Xia et al., 2020). For example, the overexpression of SARP family regulatory gene *pieR* in *Streptomyces piomogeues* var. *hangzhouwanensis* enhances the yield of piericidin A1 by 2.3-fold (Li et al., 2019); while overexpression of *otcR* considerably enhanced oxytetracycline production in *Streptomyces rimosus* by 6.49 times compared to that in the parental strain (Yin et al., 2015). Therefore, in the present study, overexpression of these two positive regulators alb22 and alb45 offers promising genetically modified strains to produce new antifoulants and further promotes the use of albofungins for antifouling application. Moreover, the albofungin yield can be further improved via the co-overexpression of alb22 and alb45 activators, as well as the overexpression of transporters to facilitate the export of the self-produced secondary metabolites.

Subsequently, fermentation of high-yield albofungin strain could easily produce gram-scale albofungin for the preparation of albofungin-based coatings. The low and constant release rate of albofungin-based coatings suggested the slow degradation rate of the copolymer that maintained the relatively constant release of the albofungin over a long period, which could hold an effective concentration longer and play an important role in the antifouling performance (Xie et al., 2019). Thus, the albofungin and PMSM0 copolymer formed a suitable coating to release albofungin in the marine field test. After 2 months of observation, albofungin inhibited macrofoulers attachment, and only a few fouling organisms have settled on the 5 and 10 wt% of albofungin-coated panels because the amount of albofungin being released from the panel into seawater decreased in the later testing stage. The result in the present study was comparable with the field performance of butenolide (Ma et al., 2017) and even better antifouling efficacy than that of DIM and DCOIT (Wang et al., 2015), which was probably due to the broader inhibitory spectrum against biofoulers of albofungin. The field test results suggested that albofungin could prevent the settlement of multiple fouling organisms in marine environments at a low concentration.

Furthermore, albofungin-based coatings changed the microbial community structure, particularly for the abundance of Proteobacteria. More specifically, the comparison of microbial diversity between the control group and the albofungin treated group demonstrated that the abundance of Alphaproteobacteria was significantly decreased, whereas the abundance of Gammaproteobacteria was increased. It was reported that the class of Alphaproteobacteria plays a crucial role in biomass accumulation and biofilm maintenance (Shelud’ko et al., 2019), which is consistent with the findings in this study. Moreover, Gammaproteobacteria was also demonstrated to be responsible for the metabolism of hydrocarbon, which is important for the defensive response to a chemical stimulus (Dyksma et al., 2016). Accordingly, the increase of Gammaproteobateria after albofungin treatment may suggest the crucial role of Gammaproteobacteria in response to albofungin treatment, implying the potential antifouling mechanism of albofungin, which deserves further investigation. Given that the biofilm can mediate the larval settlement of *H. elegans* (Chung et al., 2010), the changes in microbial community structure and biofilm density may alter the larval settlement response and could cause the inhibitive effect of the albofungin-based copolymer coatings.

The advantages of antifouling compounds isolated from marine microorganisms, such as chemical diversity and uniqueness, make them potential sources for the discovery of new antifouling compounds. These newly discovered antifoulants, however, have always been limited by low yields for commercialization (Long et al., 2021). Also, it is critical to evaluate the highly potent compounds in the field test to provide information for further marine industrial applications (Wang et al., 2017). In our study, albofungins were isolated from bacterial secondary metabolites and we constructed the high-yield strains for albofungin production in bacterial fermentation (153 ± 22.7 mg L^–1^). Our field test results suggested that albofungin had a high potential in preventing the settlement of multiple fouling organisms in marine environments at a low concentration. In terms of limitations, potent antifouling agents must be examined for their acute and chronic toxicity to non-target organisms as well as their degradation kinetics in the marine environment before commercialization (Wang et al., 2017). In the future, more research needs to be conducted on degradation kinetics and potential ecological risks investigations of albofungins.

## Conclusion

Novel antifouling agents are needed to address the issues caused by biofouling in the marine industry. In the present study, we identified potent antibiofilm and antifouling compounds albofungins isolated from the metabolites of *S. chrestomyceticus* BCC 24770 with low cytotoxicity. We also constructed high-yield strains for albofungin production and developed albofungin-based antifouling coatings that prevented colonization of macrofoulers in marine environments. Our findings revealed that broad-spectrum and strong antifouling activities, relatively low cytotoxicity, and high yield in bacterial fermentation make albofungins be promising antifouling candidates and indicated their applications in the antifouling area.

## Data Availability Statement

The datasets generated for this study can be found in the open-source online data repositories hosted at National Center for Biotechnology Information, and the accession numbers are ON014504, ON014498, and PRJNA817672.

## Author Contributions

WS, WY, and JC: conceptualization. WS: formal analysis and writing—original draft. AC, P-YQ, WY, JL, and FC: writing—review and editing. WS, RW, WKY, XL, YY, SC, and YL: investigation. CM: resources and methodology. FC, P-YQ, and AC: project administration. P-YQ: funding acquisition. All authors contributed to the article and approved the submitted version.

## Conflict of Interest

The authors declare that the research was conducted in the absence of any commercial or financial relationships that could be construed as a potential conflict of interest.

## Publisher’s Note

All claims expressed in this article are solely those of the authors and do not necessarily represent those of their affiliated organizations, or those of the publisher, the editors and the reviewers. Any product that may be evaluated in this article, or claim that may be made by its manufacturer, is not guaranteed or endorsed by the publisher.
