# Supplementary material for: Discovery, Yield Improvement, and Application in Marine Coatings of Potent Antifouling Compounds Albofungins Targeting Multiple Fouling Organisms
Source: Front Microbiol. 2022 Jul 7;13:906345. doi: 10.3389/fmicb.2022.906345 (PMC9300314; doi:10.3389/fmicb.2022.906345)
Supplement: Supplementary file 1 [file Data_Sheet_1.pdf]

Supplementary Material

1 Supplementary Figures

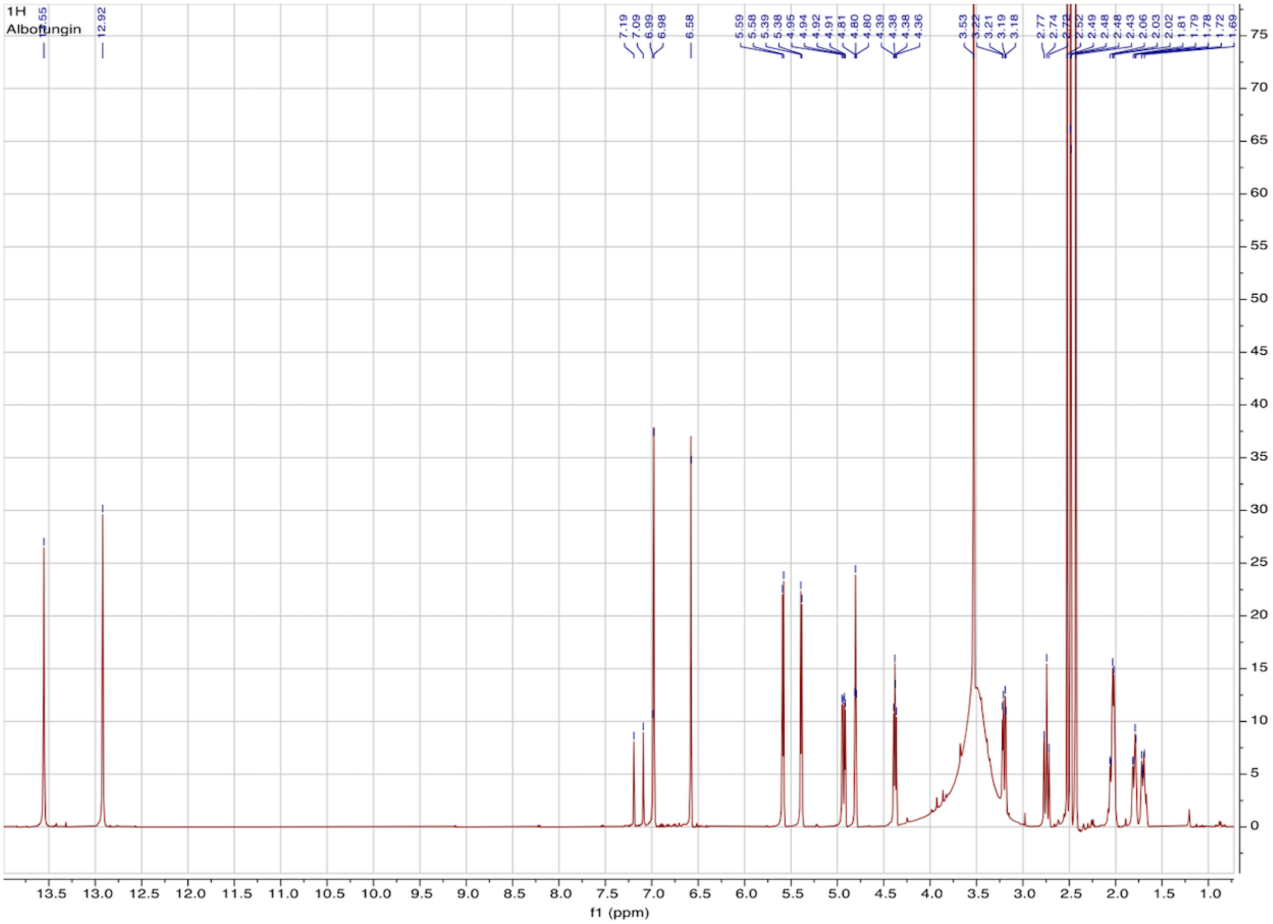

Supplementary Figure 1. ^{1}H NMR spectrum of albofungin (**1**) in DMSO-d_6.

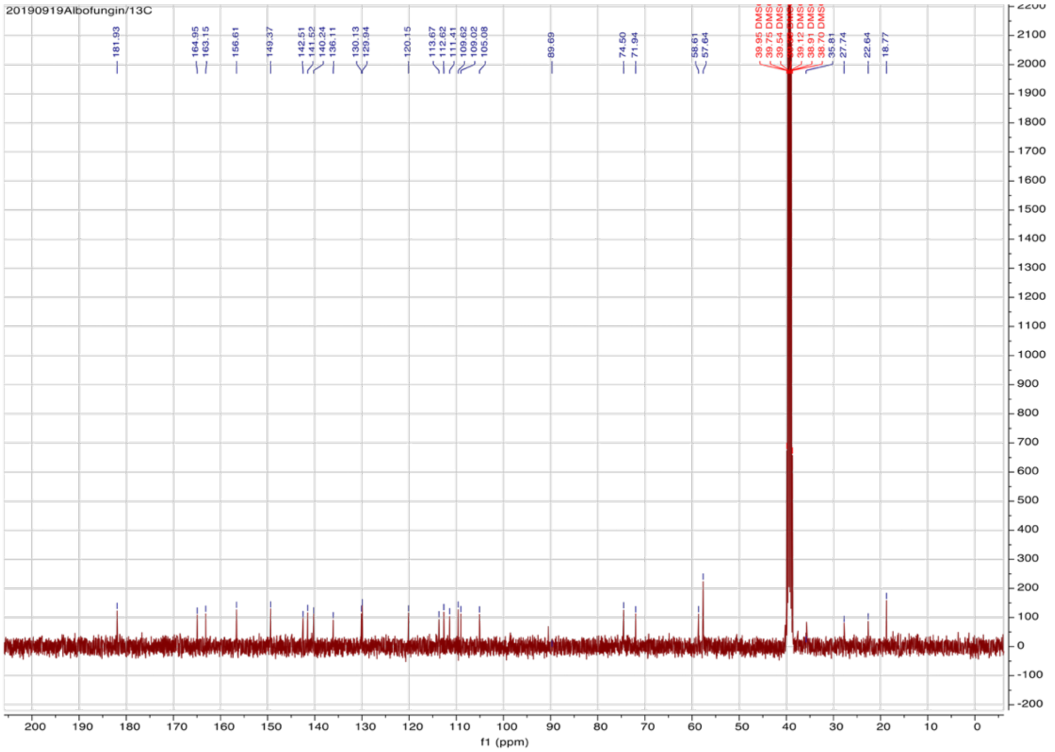

Supplementary Figure 2. ^{13}C NMR spectrum of albofungin (**1**) in DMSO-d_6.

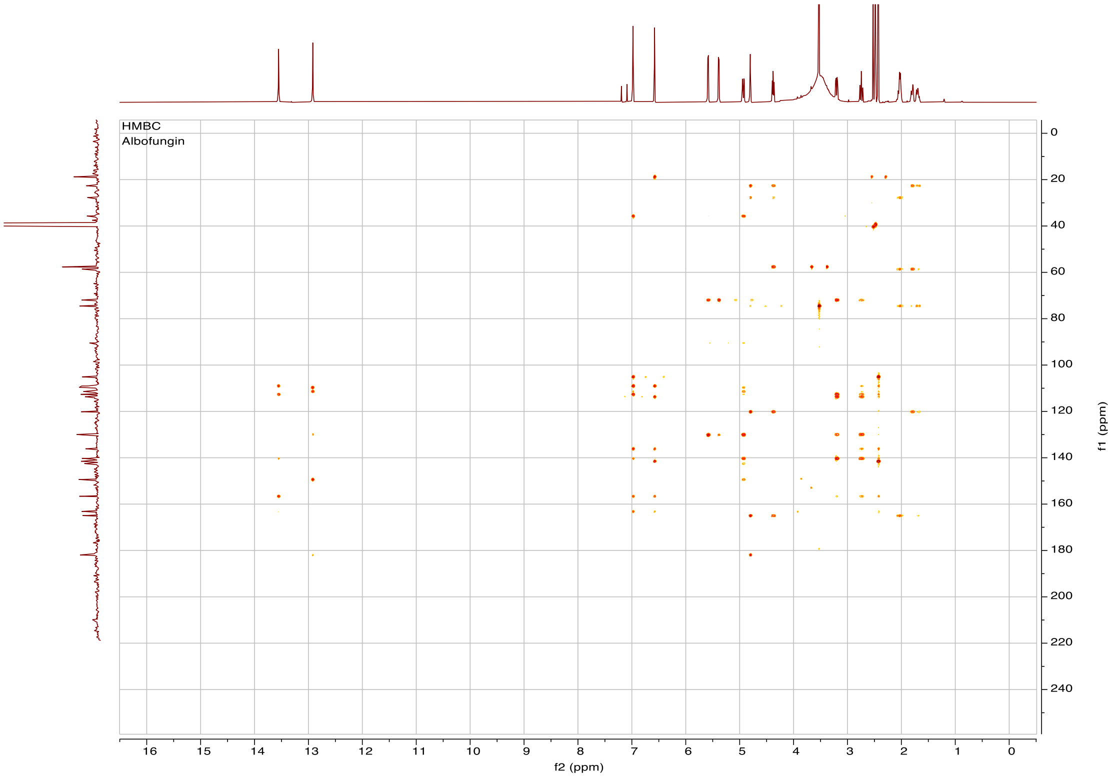

Supplementary Figure 3. HMBC spectrum of albofungin (**1**) in DMSO-d_6.

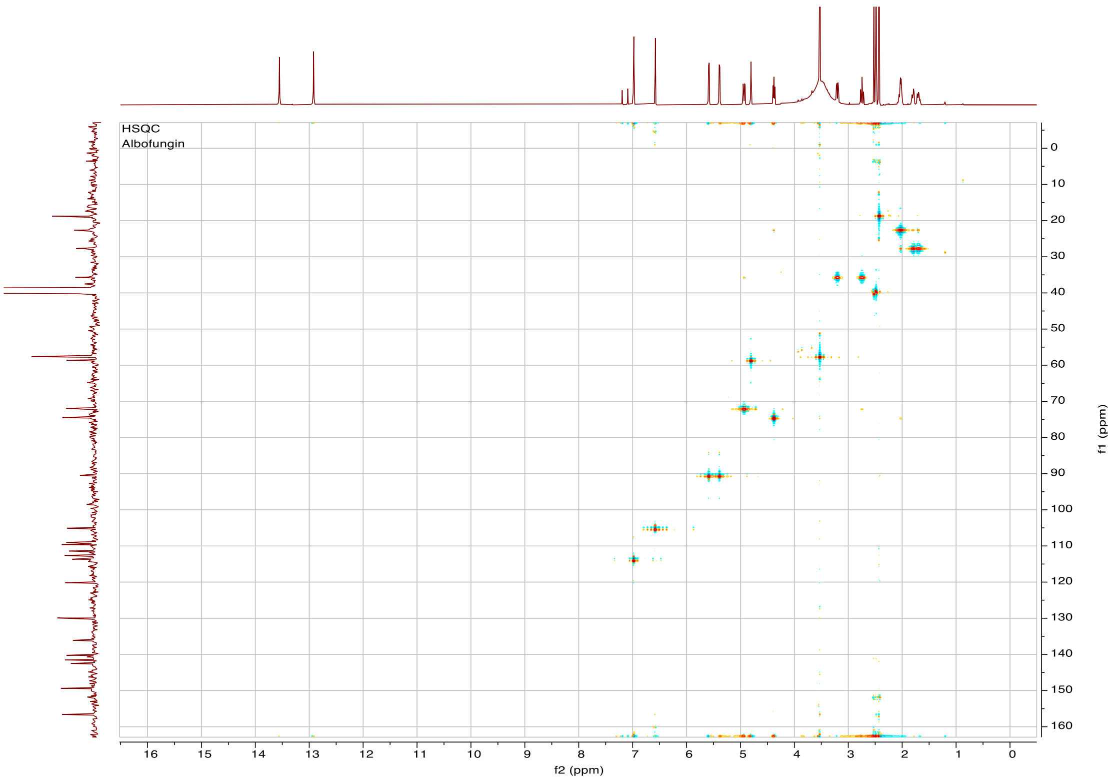

Supplementary Figure 4. HSQC spectrum of albofungin (**1**) in DMSO-d_6.

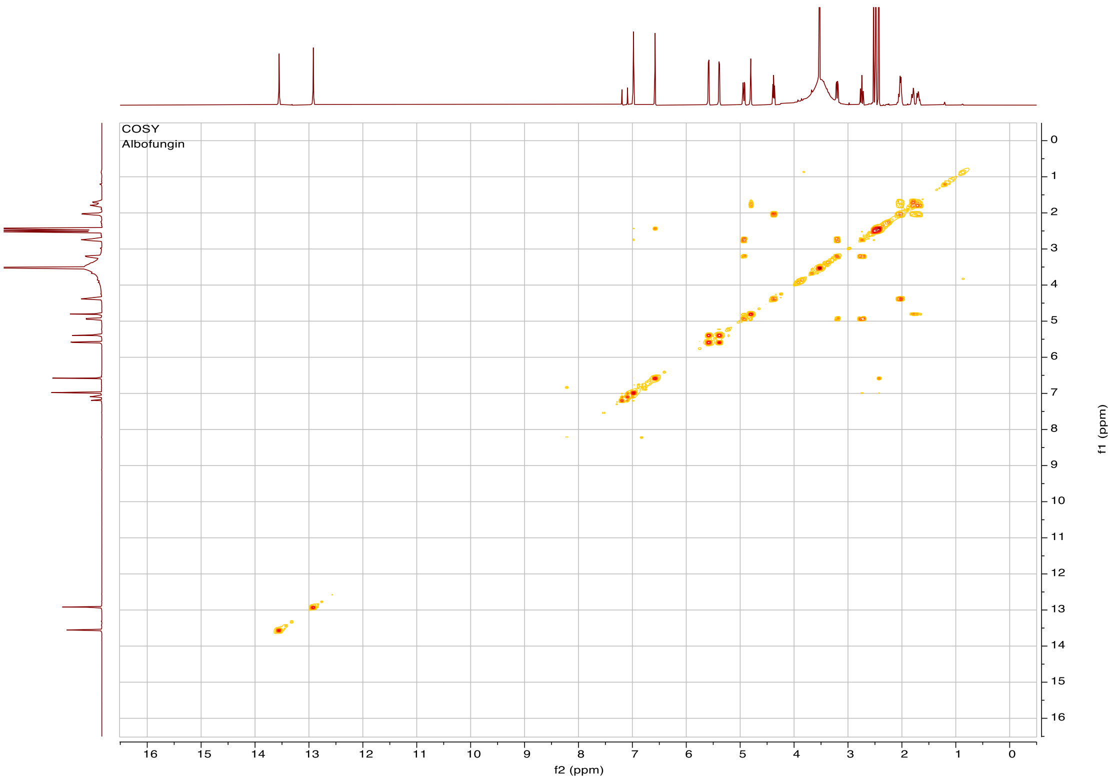

Supplementary Figure 5. COSY spectrum of albofungin (**1**) in DMSO-d_6.

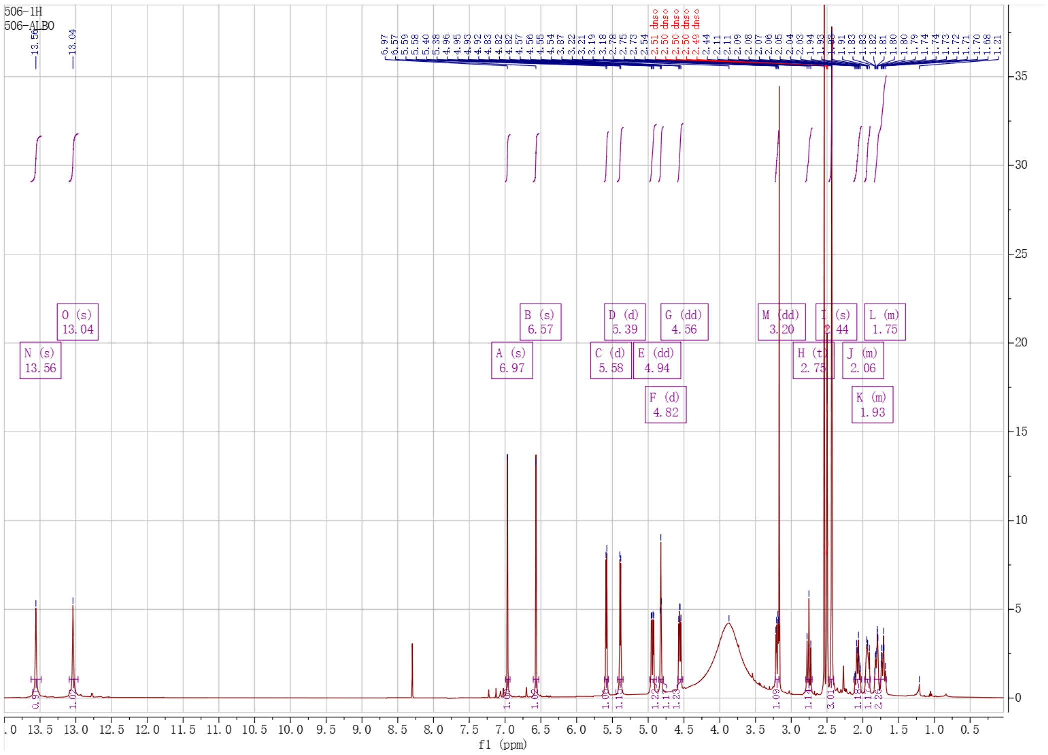

Supplementary Figure 6. ^{1}H NMR spectrum of albofungin A (**2**) in DMSO-d_6.

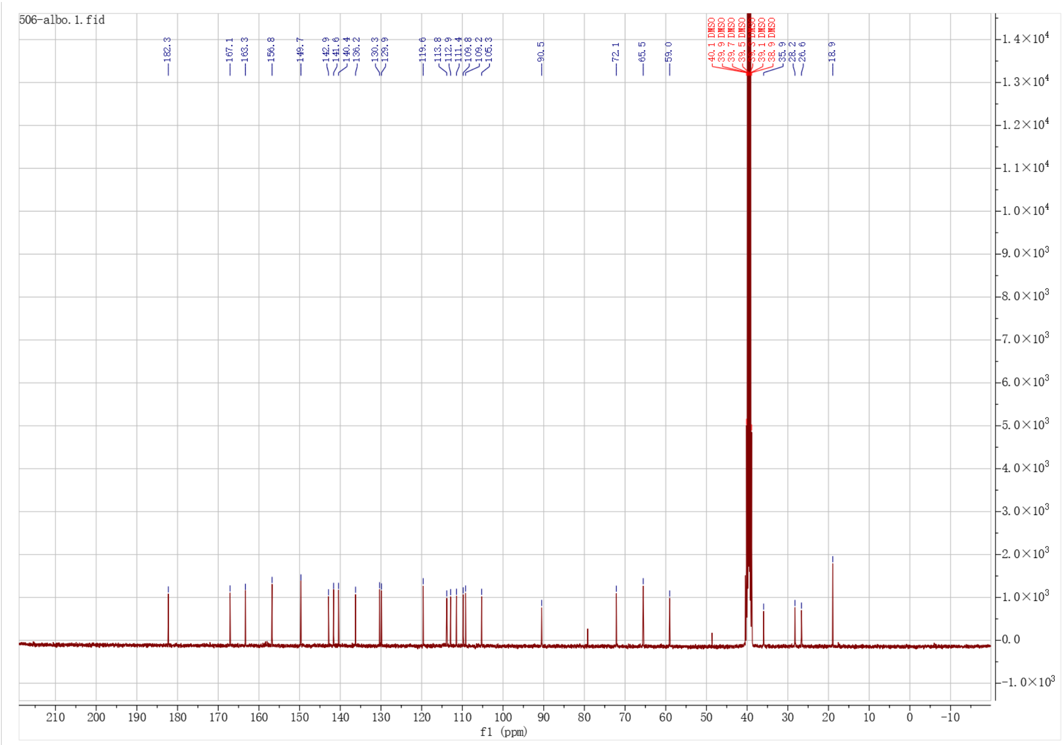

Supplementary Figure 7. ^{13}C NMR spectrum of albofungin A (**2**) in DMSO-d_6.

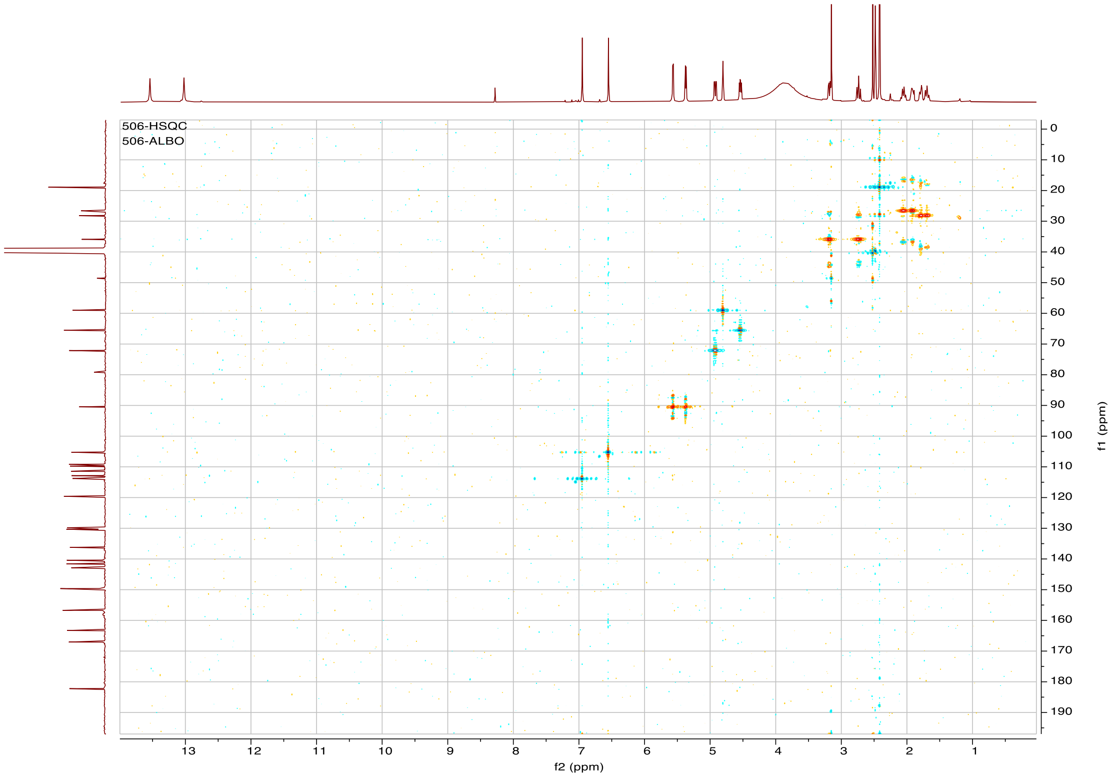

Supplementary Figure 8. HSQC spectrum of albofungin A (**2**) in DMSO-d_6.

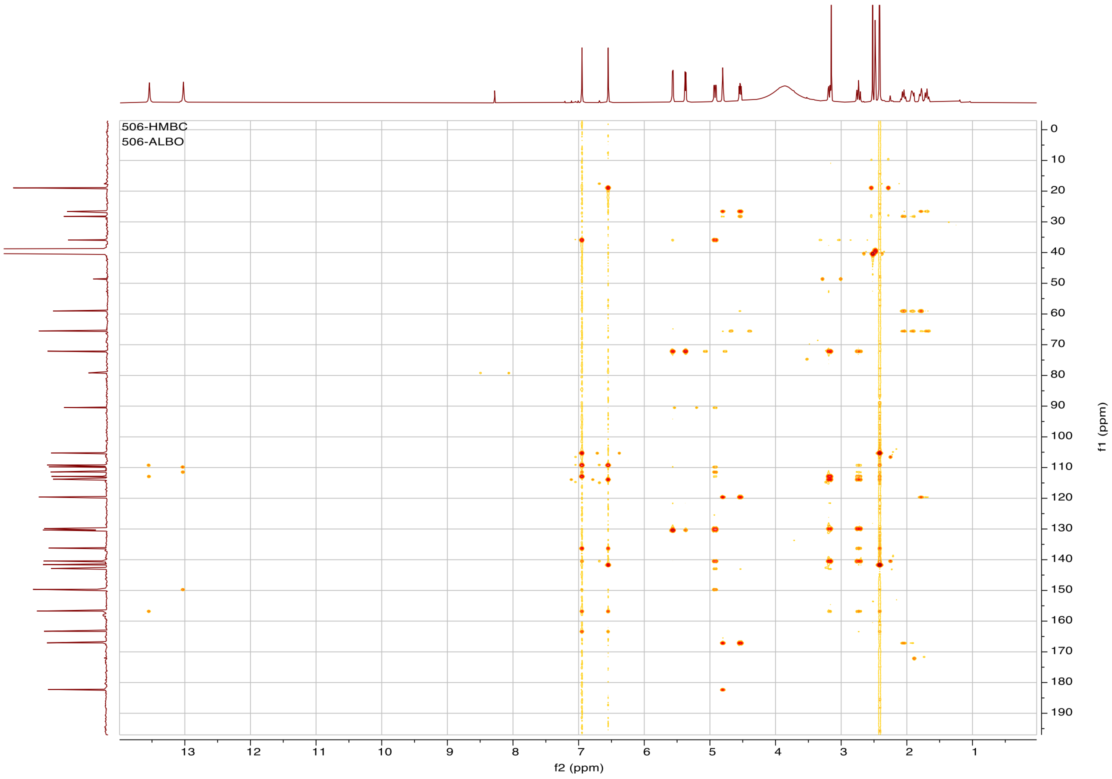

Supplementary Figure 9. HMBC spectrum of albofungin A (**2**) in DMSO-d_6

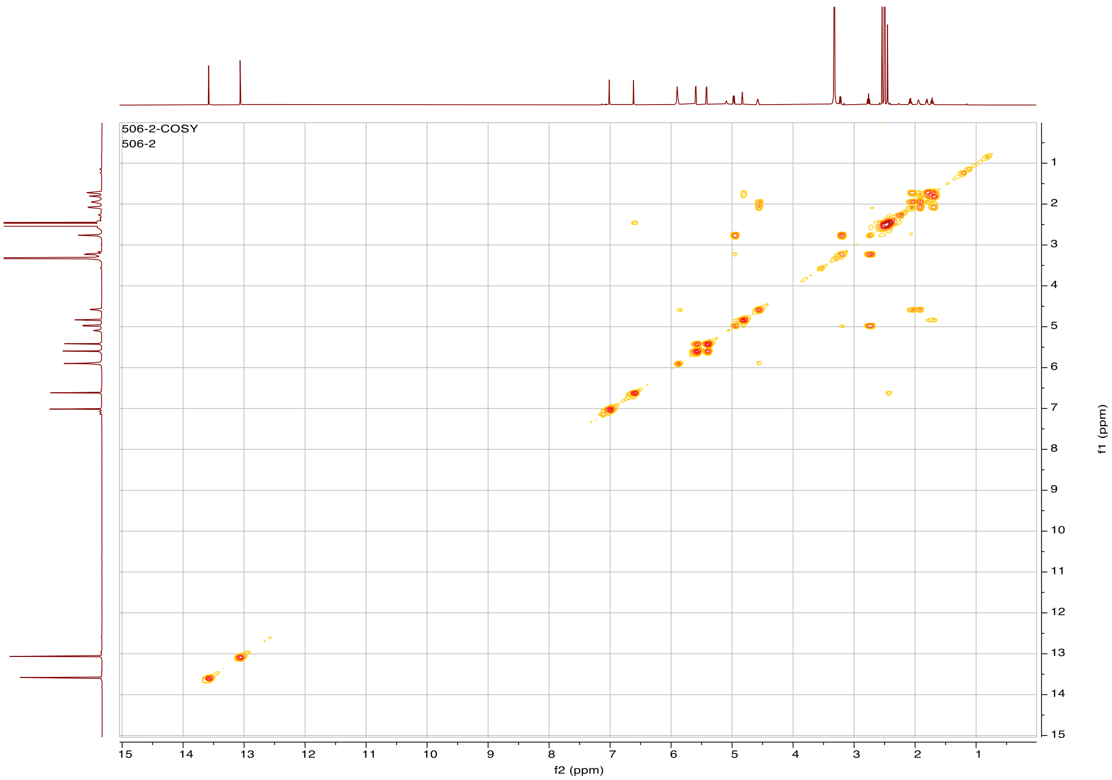

Supplementary Figure 10. COSY spectrum of albofungin A (**2**) in DMSO-d_6.

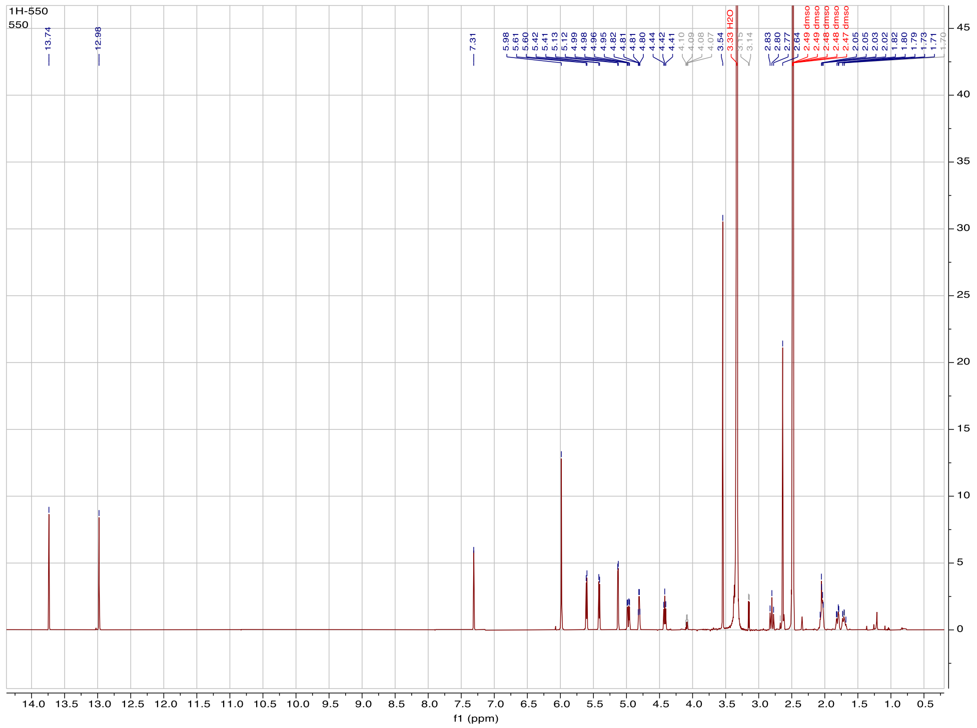

Supplementary Figure 11. ^{1}H NMR spectrum of chloroalbofungin (**3**) in DMSO-d_6.

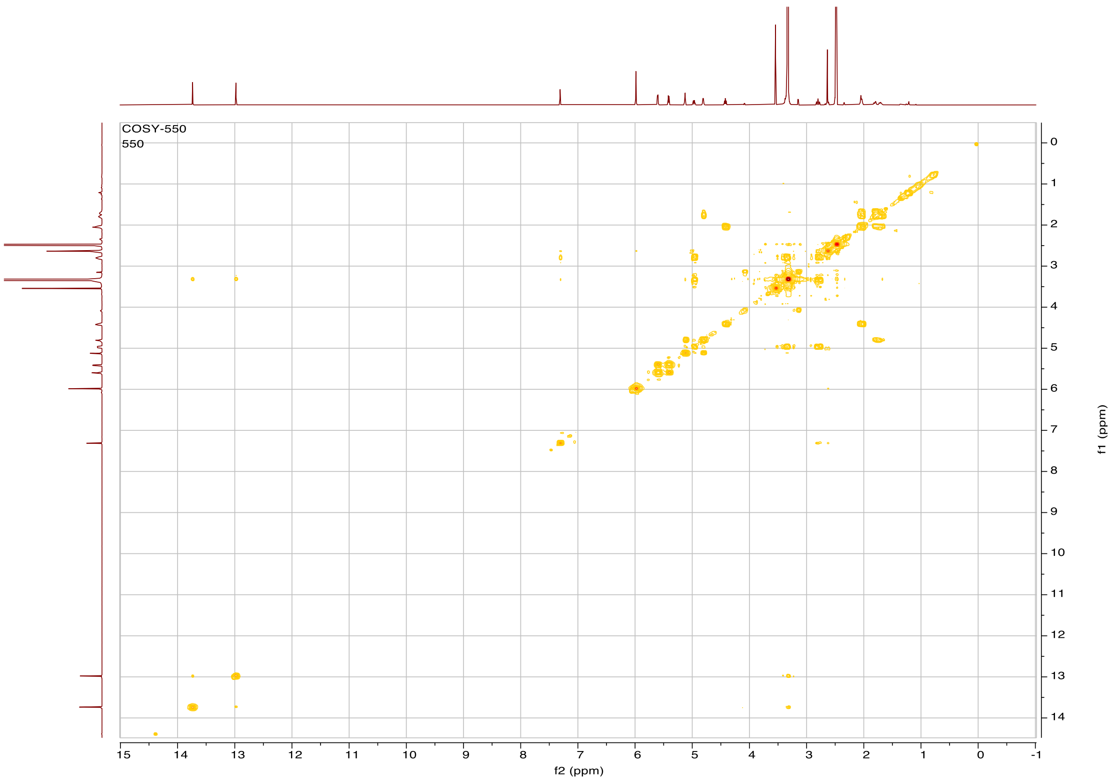

Supplementary Figure 12. COSY spectrum of chloroalbofungin (**3**) in DMSO-d_6.

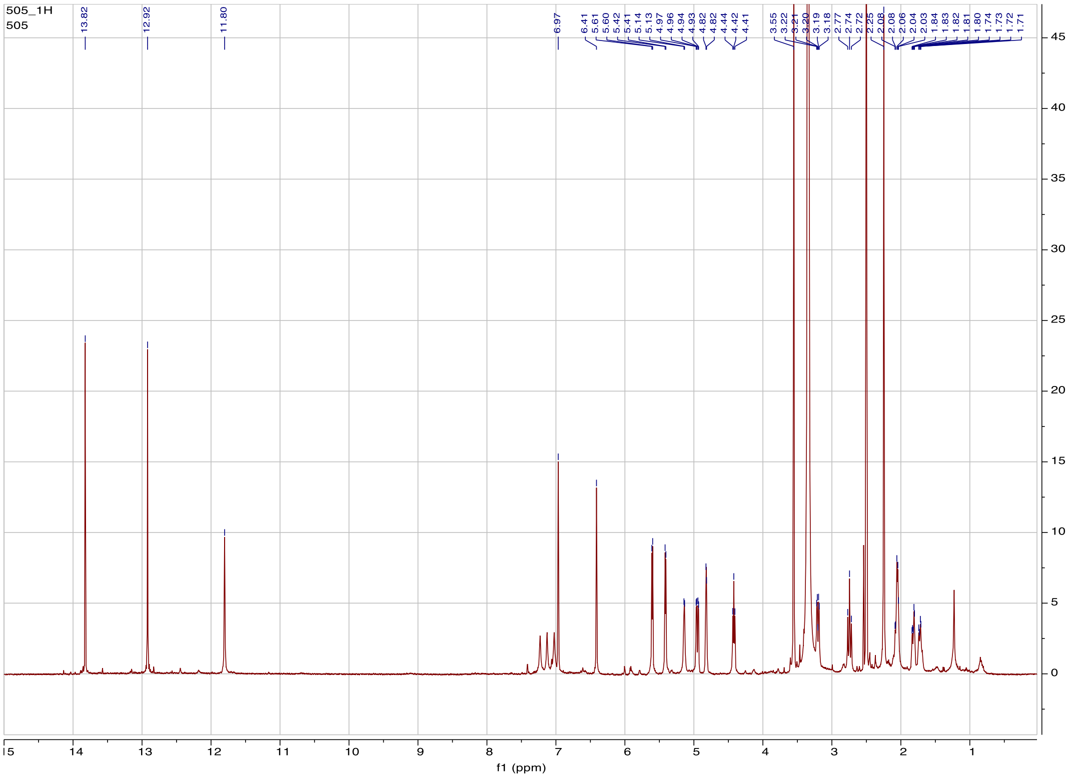

Supplementary Figure 13. ^{1}H NMR spectrum of chrestoxanthone A (**4**) in DMSO-d_6.

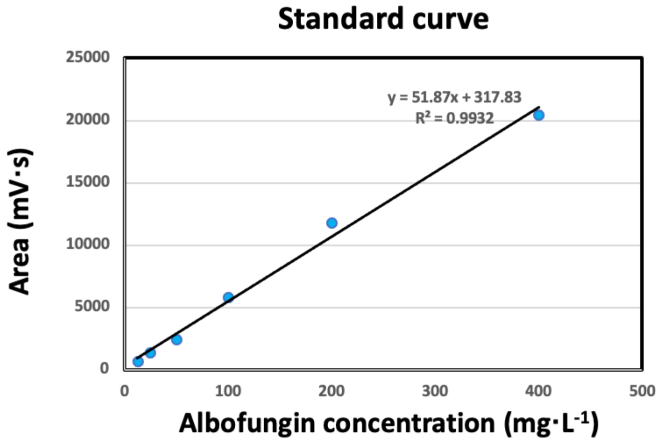

Supplementary Figure 14. Standard curve for albofungin production calculation.

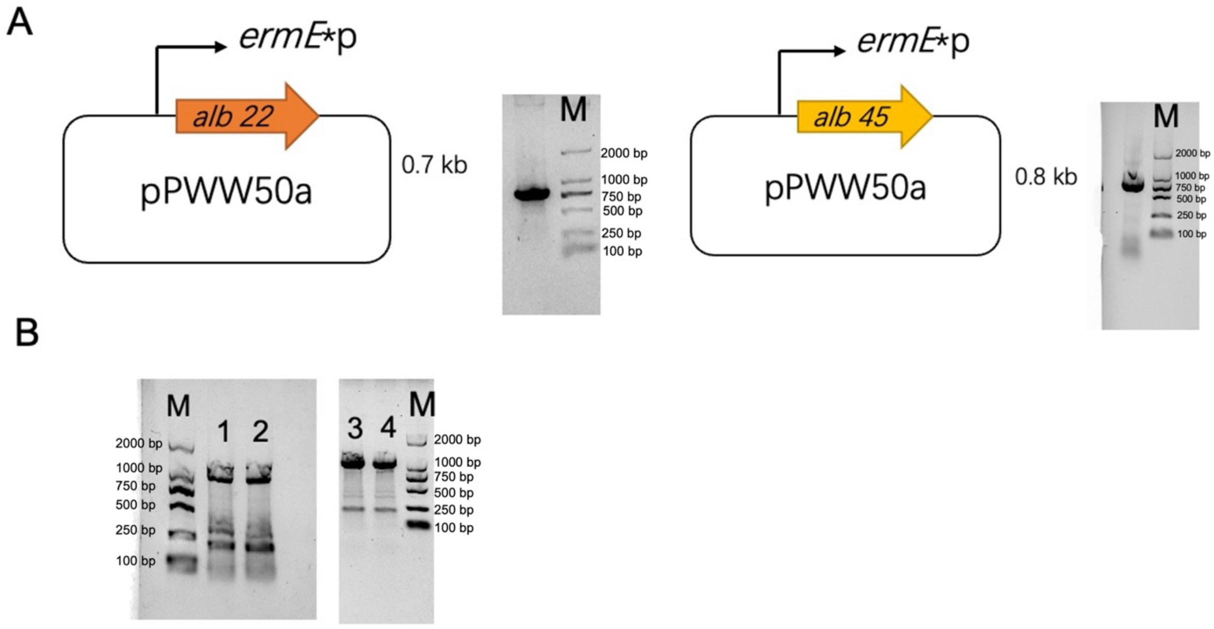

Supplementary Figure 15. (A) Construction of overexpression vectors. (B) PCR verification for the positive conjugants. M: D2000 marker; 1~2: conjugants from 24770/pPWW-*alb22*; 3~4: conjugants from 24770/pPWW-*alb45*.

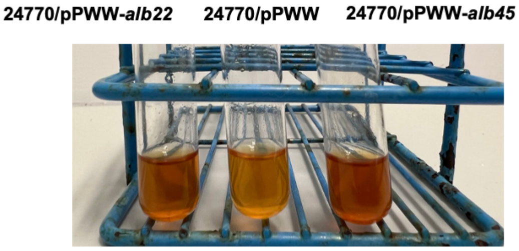

Supplementary Figure 16. Crude extracts of 24770/pPWW-*alb22*, 24770/pPWW and 24770/pPWW-*alb45* at 7 days (dissolved in methanol).

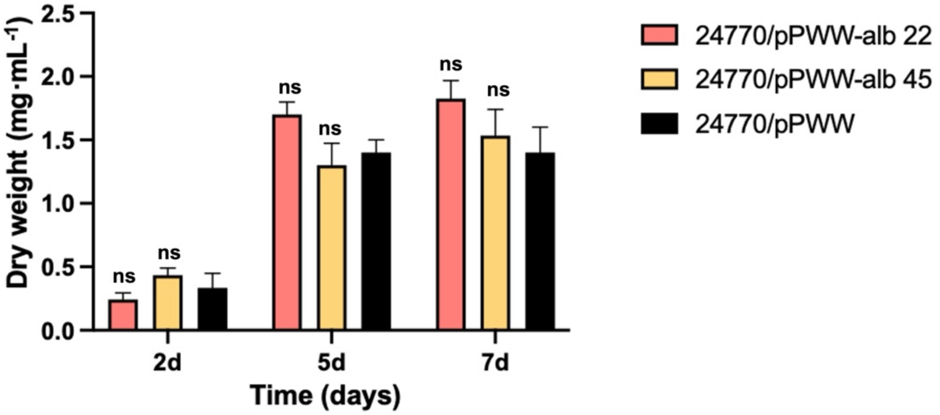

Supplementary Figure 17. Dry weight of the bacteria culture. Data are presented as mean ± standard deviation from three independent experiments. Significant differences were analysed by one-way ANOVA. ns: not significant.

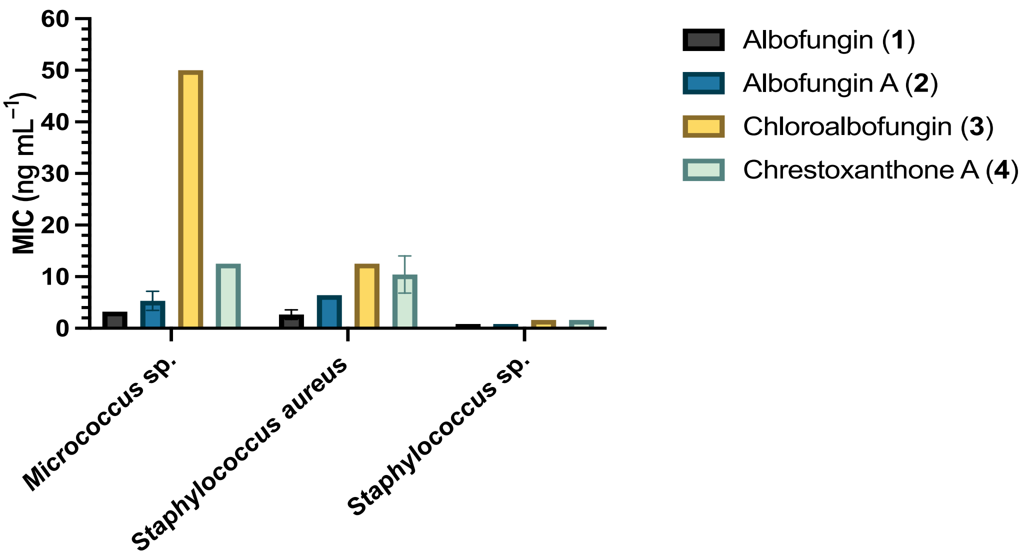

Supplementary Figure 18. Bacterial growth inhibition of albofungins (**1**–**4**) towards Gram-positive marine bacteria. Data are presented as mean ± standard deviation from three independent experiments.

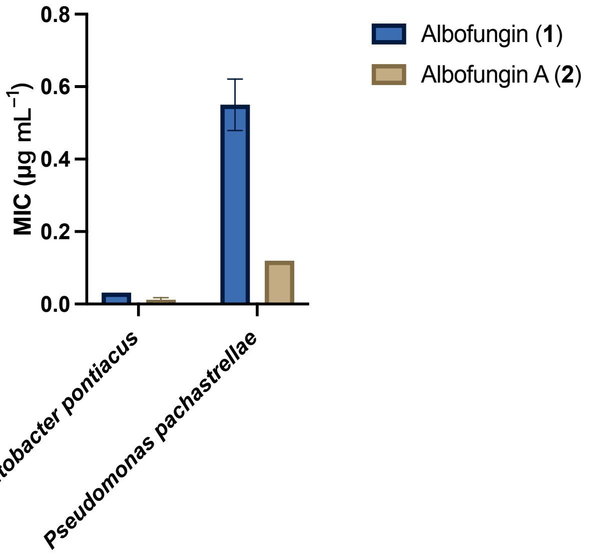

Supplementary Figure 19. Bacterial growth inhibition of albofungins (**1** and **2**) towards Gram-negative marine bacteria. Data are presented as mean ± standard deviation from three independent experiments. No bacterial growth inhibition was observed of albofungins (**3** and **4**) at 20 µg mL^{-1} against *Sulfitobacter pontiacus*, *Psychrobacter nivimaris*, and *Pseudomonas pachastrellae*; of albofungins (**1** and **2**) at 20 µg mL^{-1} against *Psychrobacter nivimaris*.

2 Supplementary Tables

Table S1. Primers used in this study.

Primers	Sequence (5'-3')
pPWW50a-alb22-F	GGAATTCCATatggagcgcgacacc
pPWW50a -alb22-R	GACTAGTtcagaacggccaggtctcct
pPWW50a-alb45-F	GGAATTCCATatggacatcagcgtactggg
pPWW50a -alb45-R	GACTAGTtcagccggcccggaccag
qPCR-GAPDH-F	TCTTCACCAAGAAGGCCGAC
qPCR-GAPDH-R	TACTTGTCCTGGTTGACGCC
qPCR-alb45-F	GTCTCGACCTCCTCACTGGT
qPCR-alb45-R	GTGCGCAGTTGGAGGATGTA
qPCR-alb22-F	GTCAGCAGTTCGTCGGTGAC
qPCR-alb22-R	CCCACCACACTCGGCTACAC
pPWW50a -check-F	AACCATGAGAGGAGAGCGGG
pPWW50a -check-R	TGAGCGAGGAAGCGGAAGAG

Table S2. EC_{50} and LC_{50} of albofungins (**1**–**4**) against barnacle *A. amphitrite* and bryozoan *B. neritina l*arvae. The significant differences were analysed by one-way ANOVA, $*p < 0.05$, $**p < 0.01$, and $***p < 0.001$.

Compound	*A. amphitrite* cyprid larvae		
	EC_{50} (µg mL^{-1})	LC_{50} (µg mL^{-1})	LC_{50}/EC_{50}
Albofungin (**1**)	1.9***	>100	52.6
Albofungin A (**2**)	9.9**	>100	10.1
Chloroalbofungin (**3**)	35.0*	>100	-
Chrestoxanthone A (**4**)	>36.8	>100	-
Butenolide	0.9***	>50	55.6
**Compound**	***B. neritina* larvae**		
	EC_{50} (µg mL^{-1})	LC_{50} (µg mL^{-1})	LC_{50}/EC_{50}
Albofungin (**1**)	1.2***	>100	83.3
Butenolide	0.5***	>50	100
